# Precocene II, a Trichothecene Production Inhibitor, Binds to Voltage-Dependent Anion Channel and Increases the Superoxide Level in Mitochondria of *Fusarium graminearum*

**DOI:** 10.1371/journal.pone.0135031

**Published:** 2015-08-06

**Authors:** Tomohiro Furukawa, Naoko Sakamoto, Michio Suzuki, Makoto Kimura, Hiromichi Nagasawa, Shohei Sakuda

**Affiliations:** 1 Department of Applied Biological Chemistry, Graduate School of Agricultural and Life Sciences, The University of Tokyo, Tokyo, Japan; 2 Department of Biological Mechanisms and Functions, Graduate School of Bioagricultural Sciences, Nagoya University, Aichi, Japan; Soonchunhyang University, REPUBLIC OF KOREA

## Abstract

Precocene II, a constituent of essential oils, shows antijuvenile hormone activity in insects and inhibits trichothecene production in fungi. We investigated the molecular mechanism by which precocene II inhibits trichothecene production in *Fusarium graminearum*, the main causal agent of Fusarium head blight and trichothecene contamination in grains. Voltage-dependent anion channel (VDAC), a mitochondrial outer membrane protein, was identified as the precocene II-binding protein by an affinity magnetic bead method. Precocene II increased the superoxide level in mitochondria as well as the amount of oxidized mitochondrial proteins. Ascorbic acid, glutathione, and α-tocopherol promoted trichothecene production by the fungus. These antioxidants compensated for the inhibitory activity of precocene II on trichothecene production. These results suggest that the binding of precocene II to VDAC may cause high superoxide levels in mitochondria, which leads to stopping of trichothecene production.

## Introduction

**Fig 1:**
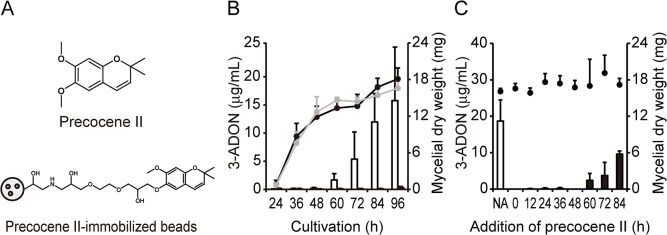
Precocene II-mediated inhibition of 3-ADON production by *F*. *graminearum*. (A) Precocene II (upper) and precocene II-immobilized magnetic beads (lower). (B) Time courses of fungal growth and 3-ADON production, and the effect of precocene II on 3-ADON production. *F*. *graminearum* was cultured with or without precocene II (30 μM). 3-ADON concentrations in the culture filtrate and the mycelial weight were analyzed. White bars, 3-ADON concentration (control); black bars, 3-ADON concentration (with precocene II); black dots, mycelial dry weight (control); gray dots, mycelial dry weight (with precocene II). (C) Effects of precocene II on 3-ADON production at different addition times. *F*. *graminearum* was cultured for 96 h. 3-ADON concentrations in the culture filtrate and the mycelial weight were analyzed. Precocene II (30 μM final concentration) was added at each cultivation time indicated in the figure during cultivation of the fungus. Bars, 3-ADON concentration; black dots, mycelial dry weight. In NA, precocene II was not added. Error bars indicate standard deviation (SD) of *n* = 6 (B), *n* = 3 (C) biological replicates.

The compound in Fig 1A was named ageratochromene when first identified as a constituent of essential oils of *Ageratum* species [1]. Later, the compound became known as precocene II after its specific antijuvenile hormone activity in insects was discovered [2]. Despite the importance for developing insect growth regulators, the target molecule of precocene II in insects has yet to be identified.

Recently, we found that precocene II inhibits trichothecene production by the fungus *Fusarium graminearum* without inhibiting fungal growth [3]. *F*. *graminearum* is the predominant plant pathogen in Fusarium head blight and produces trichothecene mycotoxins, such as deoxynivalenol, in infected grains [4]. Trichothecene contamination in major cereal crops is a very serious problem because of its impact on human and animal health and the economy. Specific inhibitors of trichothecene production, such as precocene II, are useful for controlling trichothecene contamination without incurring the rapid spread of resistant strains [5]. Such inhibitors are also useful as probes to investigate the basic regulatory mechanism of trichothecene production. Understanding this regulatory mechanism is very important for determining the optimal target of methods to control trichothecene contamination. As such, we have been investigating the mode of action by which precocene II inhibits trichothecene production.

Trichothecenes are biosynthesized from farnesyl pyrophosphate produced through the mevalonate pathway [6], in which mevalonate is the key intermediate biosynthesized from three acetyl-CoA molecules. Juvenile hormones are also biosynthesized from farnesyl pyrophosphate or its derivatives [7]. Trichothecene biosynthesis from farnesyl pyrophosphate is controlled by TRI proteins encoded by *Tri* genes [6]. Among them, TRI6 acts as a key transcription factor for trichothecene biosynthesis in *F*. *graminearum* [6, 8, 9]. TRI6 induces the expression of *Tri* genes including *Tri6* itself and upregulates the expression of genes encoding mevalonate pathway enzymes [8, 9]. Overall, the biosynthetic pathway from acetyl-CoA to trichothecenes is under control of TRI6. Upstream events that induce *Tri6* expression have not been verified, and sufficient supply of acetyl-CoA may be required for trichothecene biosynthesis.

The *F*. *graminearum* strain MAFF101551 produces 3-acetyldeoxynivalenol (3-ADON) as the main trichothecene in liquid culture. Sucrose is a key carbon source for high-level trichothecene production by this strain [10]. In a previous study, precocene II inhibited 3-ADON production by this strain with a half-maximal inhibitory concentration (IC_50_) of 1.2 μM without affecting fungal growth. This inhibition was achieved by reducing the mRNA levels of *Tri6* and genes under the regulation of TRI6 [3, 11]. Precocene II also reduced the expression of ATP citrate lyase (ACL), which is responsible for production of acetyl-CoA in the fungal cytosol [12], and the amount of acetyl-CoA in the fungal cells [13]. The citrate molecule used for ACL is supplied by the mitochondria. These results suggest that precocene II targets the regulatory pathway leading to expression of *Tri6* and *ACL*.

Some histological studies have shown that precocene II affects mitochondrial function and structure. In insects, treatment with precocene II led to mitochondrial degeneration and mitochondrial ultrastructural changes in the bug *Oncopeltus fasciatus* [14] and the cockroach *Blattella germanica* [15]. In rat hepatocytes, precocene II caused early loss of mitochondrial membrane potential [16]. In the fungus *Aspergillus flavus*, treatment of an essential oil containing precocene II as a major constituent degraded mitochondria [17]. These observations suggest that precocene II may attack mitochondria directly in these organisms.

In this paper, we describe the identification of a precocene II-binding protein in *F*. *graminearum* and investigate the molecular mechanism by which precocene II inhibits trichothecene production. Voltage-dependent anion channel (VDAC), a mitochondrial outer membrane protein, was identified as a precocene II-binding protein, and superoxide was determined to be a key molecule for trichothecene production in the fungus.

## Results

### Identification of a precocene II-binding protein

Fig 1B shows the time course of 3-ADON production by *F*. *graminearum* MAFF101551. Fig 1B and 1C show the effects of precocene II on 3-ADON production by the fungus. 3-ADON production was initiated at 60 h of cultivation. Addition of precocene II (30 μM) at the beginning of cultivation inhibited 3-ADON production almost completely throughout cultivation up to 96 h (Fig 1B). *F*. *graminearum* was cultured for 96 h while adding precocene II at each cultivation time indicated in Fig 1C. When precocene II was added at 12, 24, 36, or 48 h of cultivation, 3-ADON production was inhibited almost completely, similarly to the case when precocene II was added at 0 h. When precocene II was added at 60, 72, or 84 h, the amount of 3-ADON did not seem to increase after each addition. This result indicates that precocene II has an immediate inhibitory effect on 3-ADON production, and that the target of precocene II may be present constitutively in the fungal cells.

To identify the precocene II-binding protein, protoplasts of the mycelia cultured for 48 h were roughly fractionated to mitochondrial, cytosolic, and nuclear/unbroken cell fractions, and protein extracts from each fraction were prepared. Mitochondrial enrichment in the mitochondrial fraction was confirmed by detection of cytochrome c by immunoblotting (S1 Fig). Precocene II-immobilized magnetic nanobeads (Fig 1A) were prepared by coupling a phenolic alcohol of 6-*O*-demethylprecocene II with an epoxide of a commercially available ferriteglycidyl methacrylate bead (S1 Scheme) [18, 19]. Precocene II-immobilized beads were incubated with each protein extract mentioned above and magnetically collected. Beads were washed three times with buffer. Beads-binding proteins were eluted with precocene II.

**Fig 2:**
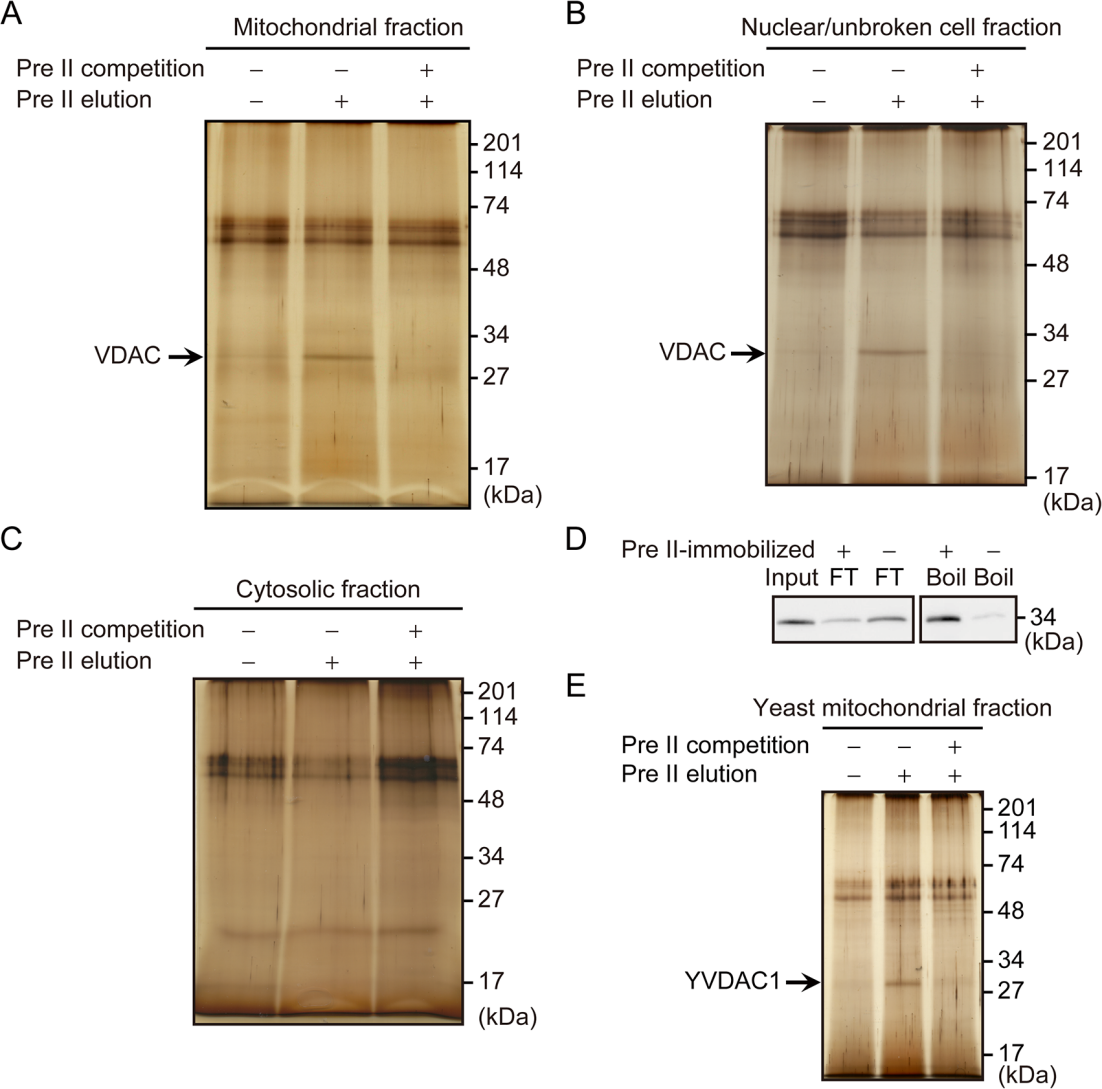
Binding experiments with precocene II-immobilized magnetic beads. Precocene II-binding protein was purified by using precocene II-immobilized magnetic beads with protein extracts from the mitochondria fraction (A), nuclear/unbroken cell fraction (B), and cytosolic fraction (C) of *F*. *graminearum* protoplasts, as well as from the mitochondria fraction of *S*. *cerevisiae* (E). Each protein extract was incubated with the beads, which were collected, washed three times with buffer, and eluted with a solution containing free precocene II. Pre II elution (−), third washing fraction; Pre II elution (+), free precocene II eluting fraction; Pre II competition, precocene II was added to protein extract before incubation with the beads. Separated proteins were visualized by silver staining. (D) Experiment with recombinant His-VDAC. Purified recombinant His-VDAC was incubated with precocene II-immobilized or control beads. Bead-bound proteins were eluted by boiling. His-VDAC was detected with anti-His antibody. FT, flow-through fraction (bead-unbound proteins).

Fig 2A–2C show the sodium dodecyl sulfate-polyacrylamide gel electrophoresis (SDS-PAGE) results of the washed and precocene II-eluted fractions. In the experiment with protein extract from the mitochondrial fraction, a band around 30 kDa was clearly observed in the precocene II-eluted fraction (Fig 2A). The band almost disappeared when precocene II was added to the mitochondrial protein extract before incubation with the precocene II-immobilized beads, suggesting specific binding of precocene II to a protein involved in the 30 kDa band. This band was also detected in the experiment with the nuclear/unbroken cell fraction (Fig 2B), but not in the experiment with the cytosolic fraction (Fig 2C). The proteins in the 30 kDa bands obtained in experiments with the mitochondrial and nuclear/unbroken cell fractions were subjected to tryptic digestion and liquid chromatography-tandem mass spectrometry (LC/MS/MS) analysis. VDAC, also known as mitochondrial porin, was the highest scoring candidate protein in these experiments (S1 Table and S2 Table).

A binding experiment was also performed by using the precocene II-immobilized beads and bacterially expressed recombinant His-tagged VDAC (His-VDAC). The band of His-VDAC was detected in the bead-binding fraction much more clearly than in the flow-through fraction (Fig 2D). When control beads without the precocene II moiety were used in the binding experiment, the intensity of the band in the bead-binding fraction became very weak, while the intensity of the flow-through fraction became clearer (Fig 2D).

To confirm the binding of precocene II to VDAC, precocene II-binding protein in the mitochondrial protein extract from *Saccharomyces cerevisiae* cells was analyzed by the same protocol as used for *F*. *graminearum*. The band around 30 kDa was detected in the precocene II-eluting fraction, and it disappeared in the competitive inhibition experiment with precocene II (Fig 2E). The protein involved in the band was identified as yeast channel-forming VDAC isoform (YVDAC1, S3 Table) [20]. These data strongly indicate that VDAC is the precocene II-binding protein.

### Effects of precocene II on the superoxide level

VDAC is an abundant protein located in the mitochondrial outer membrane of eukaryotes. VDAC has a role in metabolic transport across the mitochondrial outer membrane [21] and may be concerned with the release of superoxide (O_2_^.-^) from the mitochondrial intermembrane space into the cytosol [22, 23]. Superoxide in mitochondria is generated as a byproduct of the reactions in the mitochondrial electron transport chain, and some of the generated superoxide is released into the intermembrane space [22]. Paraquat, a superoxide-generating agent, has been shown to inhibit trichothecene production by *F*. *graminearum* CBS185.32 [24]. Therefore, the superoxide level in fungal cells may be an important clue for investigating the relationship between the binding of precocene II to VDAC and the inhibition of trichothecene production.

**Fig 3:**
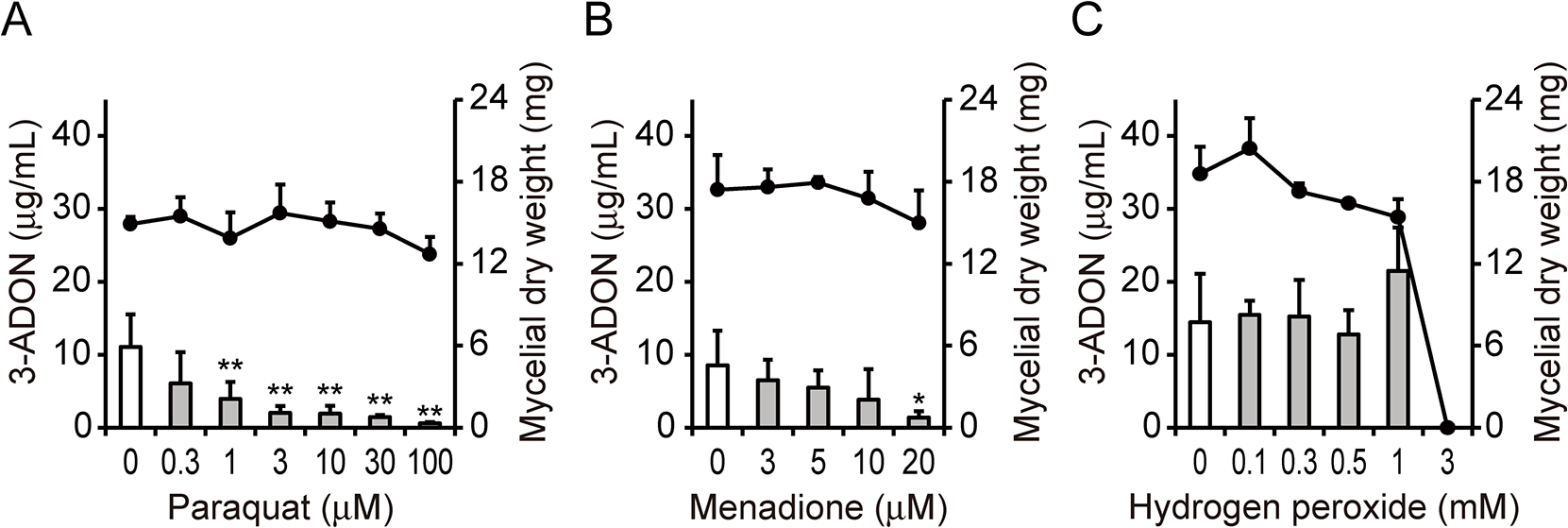
Effects of reactive oxygen species-related compounds on 3-ADON production and fungal growth. *F*. *graminearum* was cultured for 96 h with or without paraquat (A), menadione (B), or hydrogen peroxide (C) at the indicated concentrations. Bars, 3-ADON concentration; black dots, mycelial dry weight. Error bars indicate SD of *n* = 4 biological replicates. *P < 0.05, **P < 0.01 vs. control as measured by the Dunnett test.

Paraquat inhibited 3-ADON production by strain MAFF101551 used in this study in a dose-dependent manner, without affecting the fungal mycelial weight (Fig 3A), similarly to the case of strain CBS185.32. Menadione, a general superoxide-generating agent, also inhibited 3-ADON production significantly at 20 μM (Fig 3B). Superoxide is converted to hydrogen peroxide (H_2_O_2_) by superoxide dismutase. The addition of hydrogen peroxide to a culture of strain CBS185.32 at the beginning of cultivation was reported to enhance trichothecene production by the strain at a later time of cultivation [24, 25]. Therefore, the effect of hydrogen peroxide on the 3-ADON production of strain MAFF101551 was examined. When hydrogen peroxide was added at concentrations of 0.1 mM to 1 mM, 3-ADON production by the strain was not affected significantly (Fig 3C). Addition of 3 mM hydrogen peroxide inhibited fungal growth.

**Fig 4:**
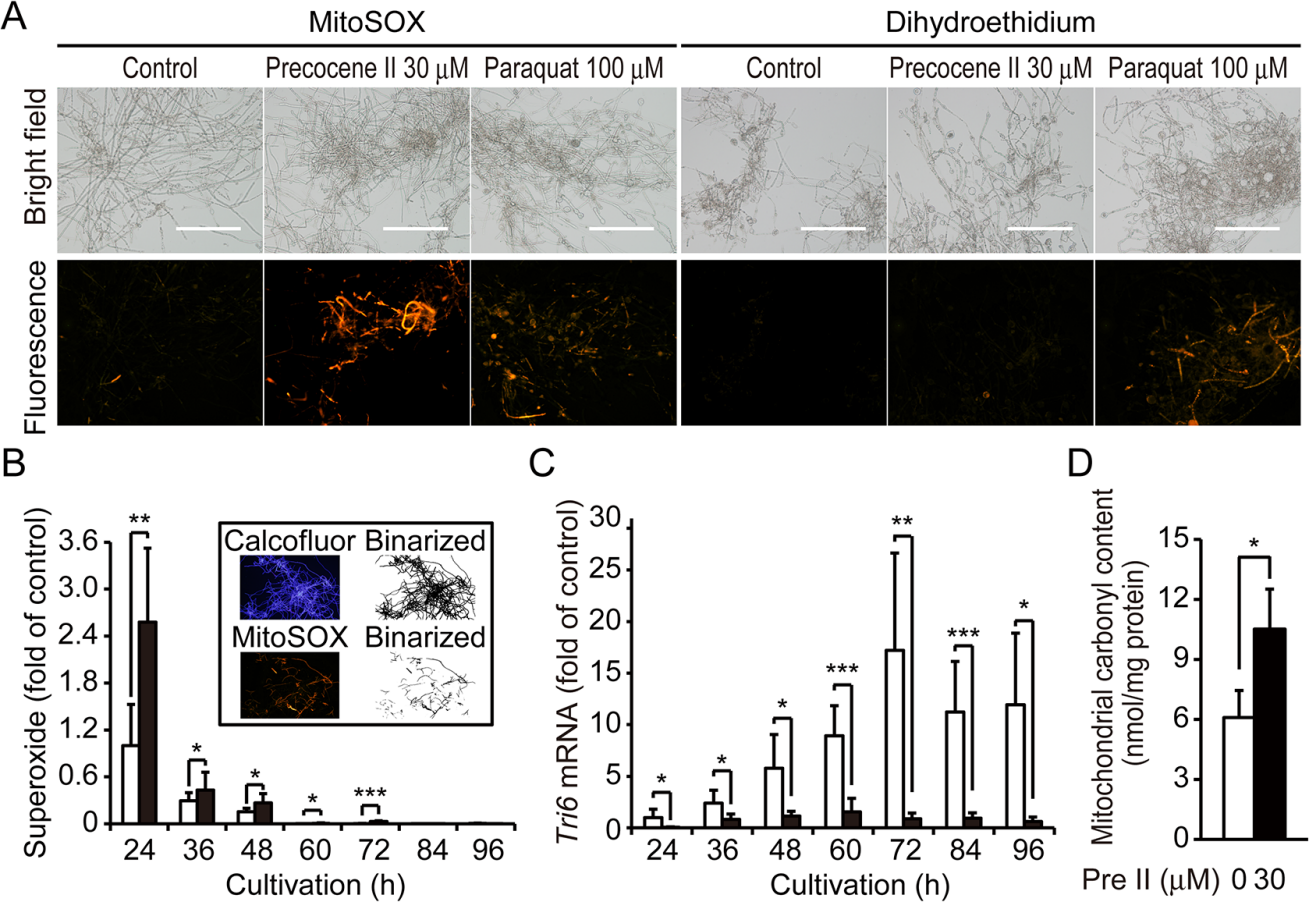
Correlation of superoxide level with inhibition of trichothecene production. (A) Precocene II (30 μM final concentration) or paraquat (100 μM final concentration) was added to a 24-h culture of *F*. *graminearum*. After cultivating the culture for another 24 h, mycelia were obtained. Superoxide in the mitochondrial and cytosolic fractions was measured by mitoSOX and DHE, respectively. Scale bars, 200 μm. (B) Time course of the mitochondrial superoxide level in fungal cells cultured with or without precocene II (30 μM). The superoxide level was roughly quantified by using mitoSOX and the calcofluor fluorescence in the microscopic photos. Upper right panel shows an example of binarized images. White bars, control; black bars, precocene II. (C) Time course of *Tri6* mRNA level of *F*. *graminearum* cultured with or without precocene II (30 μM). Amount of *Tri6* mRNA was normalized to the amount of *GAPDH* mRNA in each sample. White bars, control; black bars, precocene II. (D) Effects of precocene II (Pre II) on mitochondrial protein carbonyl content. In (B-D), precocene II was added at the beginning of cultivation, and fungi were cultivated for the indicated cultivation time (B,C) or for 96 h (D). Error bars indicate SD. In (B), data represent the means of ≥ 6 microscopic photos; *n* = 6 (C), *n* = 4 or 6 (D) biological replicates. *P < 0.05, **P < 0.01, ***P < 0.005, as measured by the Welch *t*-test (B-D).

Next, two superoxide-specific fluorescent dyes, mitoSOX red and dihydroethidium (DHE), were used to analyze the superoxide level in the cells. MitoSOX and DHE are predominantly localized to the mitochondria and cytoplasm, respectively [26]. Precocene II (30 μM final concentration) or paraquat (100 μM final concentration) was added to the fungal culture at 24 h of cultivation. After another 24 h of cultivation, obtained mycelia were treated with mitoSOX or DHE and observed under a fluorescent microscope. The intensity of mitoSOX fluorescence in the precocene II-treated hyphae was much higher than that in the control hyphae. However, the intensity of DHE fluorescence was very low in the precocene II-treated and control hyphae, indicating that precocene II increased the superoxide level in mitochondria but not in cytoplasm (Fig 4A). On the other hand, relatively high intensities of both of mitoSOX and DHE fluorescence were observed in the paraquat-treated hyphae, indicating that paraquat increased the superoxide level in both the mitochondria and cytoplasm (Fig 4A).

Fig 4B shows the time course of the mitochondrial superoxide level in the fungal cells cultured with or without precocene II. The mitochondrial superoxide level per mycelial mass was quantified by analyzing mitoSOX and calcoflour fluorescent microscopic images. The fluorescent intensity of mitoSOX and calcoflour were used as indicators of mitochondrial superoxide level and mycelial mass, respectively. For quantification, each fluorescent image was binarized and superoxide level was calculated as follows; relative superoxide level = black area of a binarized image of mitoSOX fluorescence/black area of a binarized image of calcoflour fluorescence × 100 (see example in Fig 4B). As shown in Fig 4B, the mitochondrial superoxide level in control cells without precocene II was highest at the beginning of the growth. The level gradually decreased during the growth phase to become very low at 60 h of cultivation when trichothecene production began. Addition of precocene II at the beginning of cultivation enhanced the mitochondrial superoxide level during the growth phase. The mRNA level of *Tri6* gradually increased inversely proportional to the mitochondrial superoxide level and became highest at 72 h of cultivation (Fig 4C). Precocene II suppressed expression of *Tri6* throughout the cultivation. These observations suggest that there is a relationship between a high level of superoxide in the mitochondria and inhibition of trichothecene production.

Reactive oxygen species, including superoxide and hydrogen peroxide, enhance protein oxidation which leads to increase of carbonyl groups of protein side chains [27]. Addition of precocene II (30 μM) at the beginning of cultivation significantly increased carbonyl groups on mitochondrial proteins prepared from protoplasts of the mycelia cultured for 36 h (Fig 4D). This result was consistent with the precocene II-mediated increase in mitochondrial superoxide levels that were observed under the microscope.

### Effects of antioxidants on trichothecene production and the action of precocene II

**Fig 5:**
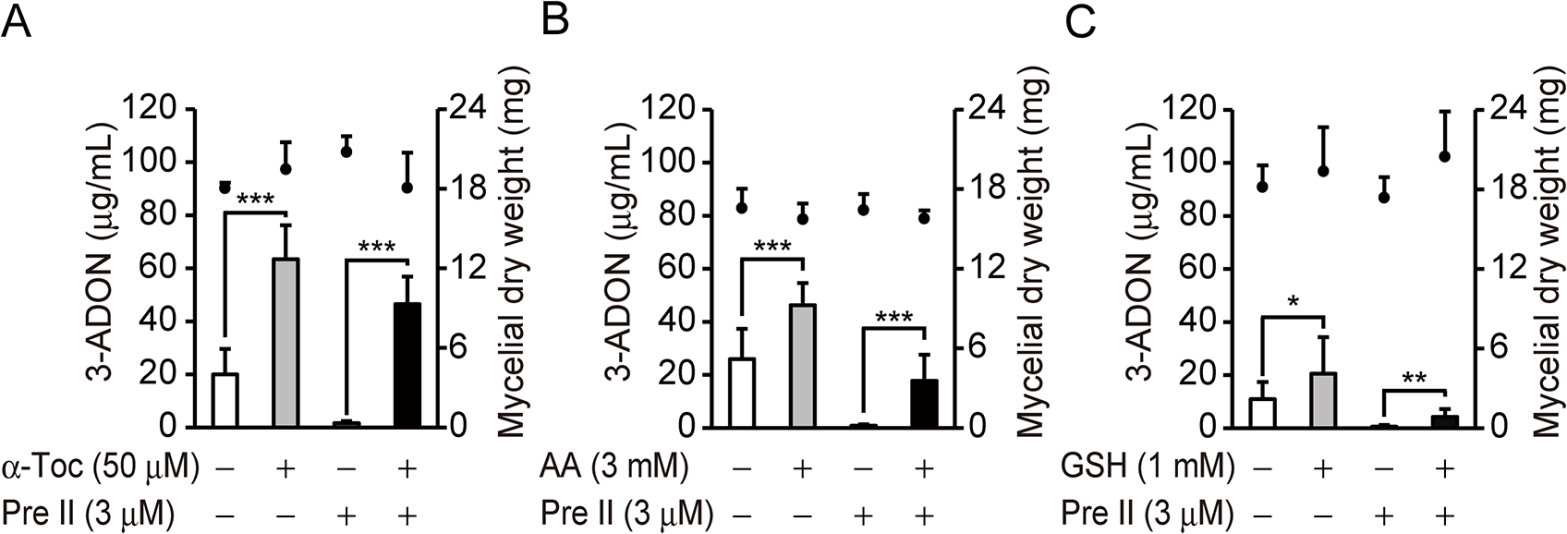
Effects of antioxidants on 3-ADON production. *F*. *graminearum* was cultured for 96 h with or without α-tocopherol (α-Toc) and/or precocene II (Pre II) (A), ascorbic acid (AA) and/or precocene II (B), or glutathione (GSH) and/or precocene II (C). Bars, 3-ADON concentration; black dots, mycelial dry weight. Error bars indicate SD of *n* ≥ 4 biological replicates. *P < 0.05, **P < 0.01, ***P < 0.005, as measured by the Welch *t*-test.

The effects of glutathione, ascorbic acid and α-tocopherol on 3-ADON production were examined. These intrinsic antioxidants are oxidized by reactive oxygen species and protect cells from oxidative stress. Addition of α-tocopherol (50 μM) at the beginning of cultivation strongly enhanced 3-ADON production, which was also enhanced by ascorbic acid (3 mM) and glutathione (1 mM) (Fig 5A–5C). Precocene II (3 μM) strongly inhibited 3-ADON production. Co-addition of α-tocopherol (50 μM) with precocene II (3 μM) compensated for the inhibitory activity of precocene II and increased the amount of 3-ADON to be near the level in the case that only α-tocopherol (50 μM) was added (Fig 5A). Co-addition of ascorbic acid (3 mM) or glutathione (1 mM) with precocene II partially compensated for the action of precocene II (Fig 5B and 5C). These results indicate that the superoxide or reactive oxygen species level may be closely related to trichothecene production by the fungus.

## Discussion

Precocene II is known for its antijuvenile hormone activity; however, its target molecule in insects has not yet been clarified. We recently identified precocene II as a specific inhibitor of trichothecene production in *F*. *graminearum* [3]. In this study, we investigated the precocene II-binding protein in the fungus. Some reports have indicated that precocene II affects mitochondria [14–17]. Therefore, we roughly fractionated protoplasts of the fungal mycelia to mitochondrial, cytosolic, and nuclear/unbroken cell fractions, and subjected the protein extracts of each fraction to affinity purification with precocene II-immobilized magnetic beads (S1 Fig and Fig 2A–2C). VDAC was identified as the precocene II-binding protein in experiments with mitochondrial and nuclear/unbroken cell fractions (S1 Table and S2 Table). VDAC is mainly expressed in the mitochondrial outer membrane, but a part of VDAC is also expressed in the plasma membrane in human [28]. In *F*. *graminearum*, expression of VDAC in the plasma membrane has not been confirmed. When VDAC is located only in the mitochondrial outer membrane in *F*. *graminearum*, detection of VDAC in the nuclear/unbroken cell fraction could be attributed to the mitochondria in unbroken cells. The presence of mitochondria in nuclear/unbroken cell fraction was suggested by detection of a small amount of cytochrome c in the nuclear/unbroken cell fraction (S1 Fig). In addition to these experiments in *F*. *graminearum*, identification of YVDAC1 as the precocene II-binding protein in the yeast *S*. *cerevisiae* and the experiment with recombinant His-VDAC supported the fact that VDAC is the bona fide binding protein of precocene II (Fig 2D and 2E and S3 Table).

In experiments with mitochondrial protein extracts of *F*. *graminearum* and *S*. *cerevisiae*, VDAC protein was eluted by free precocene II solution during purification with precocene II-immobilized beads (Fig 2A–2C and 2E). This finding indicates the specific binding of VDAC protein to the precocene II molecule. However, in the experiment with His-VDAC, free His-VDAC was not eluted from His-VDAC-bound precocene II-immobilized beads by free precocene II solution, and competitive inhibition by free precocene II was not observed. This result may suggest that His-VDAC binds very tightly to precocene II-immobilized beads. It is not clear why there is a difference between the experiments with crude protein extract containing VDAC and recombinant His-VDAC, but the control experiment using beads without the precocene II moiety clearly showed strong binding of His-VDAC to precocene II-immobilized beads (Fig 2D).

*F*. *graminearum* contains a single VDAC gene. We constructed a *vdac*-deletion mutant of *F*. *graminearum* but its growth was very poor. Hence, we could not use the mutant to confirm the importance of VDAC in the action of precocene II in an *in vivo* experiment (data not shown).

VDAC in the mitochondrial outer membrane is responsible for fluxes of ATP, NADH, and other low-molecular-weight metabolites [21, 29]. VDAC interacts with various proteins, including ATP-utilizing enzymes (e.g., hexokinase), and plays roles in the release of proapoptotic cytochrome *c*, the opening of mitochondrial permeability transition pores, and the release of superoxide from mitochondria into the cytosol [21–23]. A superoxide-generating agent, paraquat, was shown to inhibit trichothecene production [24]. Therefore, we focused on the superoxide-releasing role of VDAC to investigate the mode of action of precocene II for inhibiting trichothecene production. Precocene II was found to increase the superoxide level in mitochondria (Fig 4A).

Superoxide is generally produced as a byproduct of energy generation in the mitochondrial electron transport chain. The time course of the mitochondrial superoxide level in fungal cells suggested that a low level of mitochondrial superoxide was required for the onset of trichothecene production, because the mitochondrial superoxide decreased inversely proportional to the mRNA level of *Tri6* (Fig 4B and 4C). Antioxidants, especially α-tocopherol, enhanced trichothecene production by the fungus and counteract for the inhibitory activity of precocene II (Fig 5). Furthermore, hydrogen peroxide, which is produced by superoxide dismutase, did not affect trichothecene production significantly under our experimental conditions (Fig 3C). The effect of hydrogen peroxide on trichothecene production may depend on the strain and conditions of cultivation [30]. Taken together, these results strongly suggest that superoxide, especially mitochondrial superoxide, is a key molecule for trichothecene production.

Paraquat produces superoxide by a cyclic reduction-oxidation reaction [31]. It has been suggested that VDAC reduces paraquat to a radical form as an NADH-dependent oxidoreductase [32]. The radical produces superoxide by reducing oxygen. However, the mode of action of precocene II may be very different from that of paraquat. Whereas paraquat increased the superoxide levels in mitochondria and cytoplasm, precocene II increased the mitochondrial superoxide level exclusively (Fig 4A). It has been suggested that erastin, an oncogene-selective lethal compound, can bind to and alter the gating of human VDACs, resulting in mitochondrial dysfunction [33]. G3139, an 18-mer phosphorothioate oligonucleotide blocker of VDAC, was shown to increase accumulation of mitochondrial superoxide by binding to VDAC and blocking the efflux of superoxide from mitochondria [34]. Given these reports, we speculate that precocene II affects VDAC gating, probably gate closing, leading to increase of the superoxide level in mitochondria.

**Fig 6:**
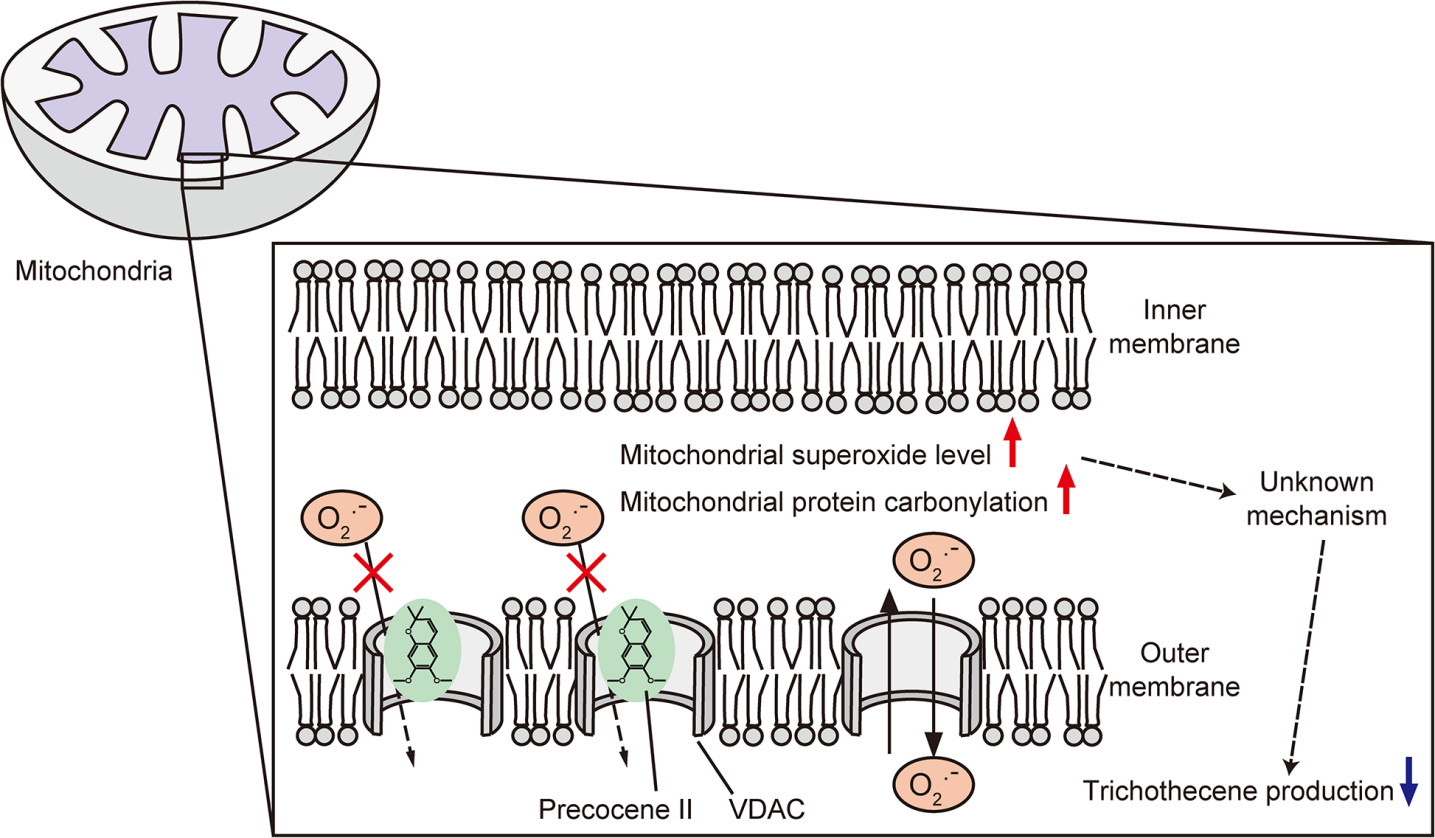
Schematic representation of the putative mechanism of action of precocene II. Precocene II binds to mitochondrial VDAC, leading to increase of mitochondrial superoxide. High-level superoxide in mitochondria may affect mitochondrial function, which results in the inhibition of trichothecene production.

How the superoxide level in mitochondria relates to trichothecene production is not clear. Because precocene II reduces the amount of acetyl-CoA in fungal cells [13], it is conceivable that interruption of mitochondrial function by high-level superoxide reduces the supply of a source of cytosolic acetyl-CoA, such as citrate, which may lead to suppression of *Tri6* expression (Fig 6).

Insect juvenile hormone is biosynthesized in the corpora allata. Ultrastructural studies suggest a correlation of mitochondrial morphology with juvenile hormone biosynthesis activity in cells of the corpora allata [35]. If precocene II acts in insects according to its mode of action in the fungus, precocene II may bind to mitochondrial VDAC in cells that are actively biosynthesizing juvenile hormones. Precocene II may increase the superoxide level in the mitochondria, leading to the inhibition of juvenile hormone production. Studies to confirm the mode of action of precocene II in insects are now in progress.

## Materials and Methods

### Reagents

Precocene II was purchased from Santa Cruz Biotechnology (Dallas, USA). 3-ADON standard was purchased from Sigma-Aldrich (St. Louis, USA). All other chemicals and solvents were purchased from Sigma-Aldrich (St. Louis, USA), Kanto Chemical (Tokyo, Japan), or Nacalai Tesque (Kyoto, Japan), unless otherwise specified.

### *F*. *graminearum* culture conditions

A Japanese isolate strain, *F*. *graminearum* MAFF101551 was used as the 3-ADON producer [13]. A spore suspension of the strain was prepared by using carnation leaf agar medium [36]. Five milliliters of liquid SYEP medium (5% sucrose, 0.1% yeast extract, and 0.1% polypeptone) were placed in test tubes (1.6 cm × 18 cm) and autoclaved. Aqueous paraquat (5 μL), DMSO-solubilized menadione (5 μL), methanolic α-tocopherol (2.5 μL), aqueous ascorbic acid (15 μL), aqueous glutathione (5 μL), aqueous hydrogen peroxide (5 μL), and/or methanolic precocene II (1.5 μL) were added to the medium (Final concentrations of these compounds were indicated in Figs 1, 3, 4 and 5. Final concentration of DMSO or methanol was 0.1% or less). Each tube was inoculated with a spore suspension of the strain (3 × 10^4^ spores/tube) and incubated with continuous shaking (300 rpm) at 26.5°C for 24–96 h. The resulting culture broth was filtered to obtain the mycelia and filtrate. Mycelia were frozen in liquid nitrogen and lyophilized to determine mycelial dry weight, and filtrate was used for analysis of 3-ADON amount, as described below.

### Analysis of 3-ADON

An aliquot of the filtrate (1 mL) above mentioned was extracted with 400 μL of ethyl acetate. Ethyl acetate solution was evaporated to dryness, and the obtained residue was dissolved in 50 μL of methanol. This solution (10 μL) was mixed with 190 μL of 10% acetonitrile in water and subjected to liquid chromatography/mass spectrometry (LC/MS) analysis in a 2695 high-performance liquid chromatography (HPLC) system (Waters, Milford, USA) equipped with Capcell-Pak C_1_ (35 mm × 4.6 mm inner diameter) and C_18_ (250 mm × 2 mm inner diameter) columns (SHISEIDO, Tokyo, Japan). The columns were eluted with a gradient of 10–80% acetonitrile in water containing 10 mM ammonium acetate over 20 min. The flow rate was 0.2 mL/min, and the retention time of 3-ADON was 16.1 min.

MS analysis was done with a Micromass ZQ system (Waters) by electrospray ionization (ESI) in positive ion mode. Spray chamber parameters were as follows: source temperature, 120°C; desolvation temperature, 350°C; cone, 30 V; desolvation gas, 600 L/h; cone gas, 50 L/h; and capillary voltage, 2800 V. MS ions were monitored in single-ion recording mode by using the extracted ion *m/z* 339 (M+H)^+^. 3-ADON amount of each sample was determined by using a standard curve obtained from standard 3-ADON solutions.

### Preparation of 6-*O*-demethylprecocene II (7-methoxy-2,2-dimethyl-2*H*-1-benzopyran-6-ol) (1)

6-*O*-demethylprecocene II (**1**) was prepared according to the method of Ohta and Bowers [37] with slight modifications (S1 Scheme A). Hydrogen peroxide (30% in water, 8.9 mL) and selenium dioxide (0.35 g) were added to a solution of 4-hydroxy-3-methoxybenzaldehyde (6 g, 39.4 mmol) in dichloromethane (100 mL) at 0°C. The reaction mixture was stirred at 0°C for 2 h and at room temperature (RT) for 6 h, filtered, and added to water (100 mL). The organic layer was washed with 10% NaHSO_3_ (100 mL) and dried over anhydrous Na_2_SO_4_. After filtration, the filtrate was treated with 6 M NaOH (20 mL) for 20 min at RT and added to water (100 mL). The aqueous layer of the mixture was adjusted to pH 4.5 and extracted with ethyl acetate. The organic layer was dried and evaporated under reduced pressure. The obtained residue was purified by silica gel column chromatography (*n*-hexane: ethyl acetate, 85:15, Wakogel C-200 [Wako, Osaka, Japan]) to give 7-methoxy-1,4-benzenediol (**2**) (1.3 g, 29.7% yield). **2**: δ_H_ (CDCl_3_, 500 MHz): 3.85 (s, 3H), 6.32 (dd, *J* = 8.25, 2.75 Hz, 1H), 6.45 (d, *J* = 2.75 Hz, 1H), 6.76 (d, *J* = 8.25 Hz, 1H).

3,3-Dimethylacrylic acid (912 mg) and polyphosphoric acid (2.204 g) were added to **2** (1.16 g, 8.3 mmol), and the reaction mixture was autoclaved at 110°C for 20 min. Saturated NaHCO_3_ (100 mL) was added to the reaction solution, which subsequently was extracted with ethyl acetate. The organic layer was dried, evaporated, and purified by silica gel column chromatography (*n*-hexane:ethyl acetate, 85:15, Wakogel C-200) to yield 2,3-dihydro-6-hydroxy-7-methoxy-2,2-dimethyl-4*H*-1-benzopyran-4-one (**3**) (567 mg, 31.3% yield). **3**: δ_H_ (CDCl_3_, 500 MHz): 1.30 (s, 6H), 2.63 (s, 2H), 3.80 (s, 3H), 5.85 (s, 1H), 6.55 (s, 1H).

LiAlH_4_ (126 mg) was added to a solution of **3** (567 mg, 2.6 mmol) in dry THF (27 mL), and the mixture was refluxed for 6 h. After 15% NaOH (2 mL) and H_2_O (100 mL) were added at 0°C, the reaction mixture was filtered and extracted with ethyl acetate. The organic layer was dried and evaporated. The obtained residue was stirred in a solution of THF (20 mL) and 4 M HCl (1 mL) for 30 min at RT. Saturated NaHCO_3_ (100 mL) was added to the reaction solution, which was extracted with ethyl acetate. The organic layer was dried and evaporated, and the obtained residue was purified by silica gel column chromatography (*n*-hexane: ethyl acetate, 85:15, Wakogel C-200) to give 6-*O*-demethylprecocene II (**1**) (390 mg, 74.2% yield). **1**: ESI-TOFMS *m/z* 205 (M−H)^−^; δ_H_ (CDCl_3_, 500 MHz): 1.42 (s, 6H), 3.85 (s, 3H), 5.49 (d, *J* = 9.46 Hz, 1H), 6.22 (d, *J* = 9.46 Hz, 1H), 6.40 (s, 1H), 6.58 (s, 1H).

### Bead preparation

FG Beads were prepared in accordance with the instructions of the manufacturer (Tamagawa Seiki, Nagano, Japan) (S1 Scheme B). FG epoxy beads (2.5 mg) were incubated with 500 μL of DMF solution containing 50 mM 6-*O*-demethylprecocene II and 35 mg of K_2_CO_3_ at 60°C for 24 h in the dark. Beads were collected by centrifugation (15,000 × *g*, 5 min, 4°C), washed twice with 50% DMF in water, washed once with water, and washed three times with 50% MeOH in water. Resulting beads were stored in 100 μL of 50% MeOH in water at 4°C.

### Preparation of proteins from *F*. *graminearum*

*F*. *graminearum* was cultured in a 500-mL Erlenmeyer flask, containing 100 mL of SYEP liquid medium, with continuous shaking (150 rpm) at 26.5°C for 48 h. The resulting culture broth was filtered to obtain mycelia, which were resuspended in 5 mL of 1.5 M aqueous NaCl solution containing 100 mg of Yatalase (TaKaRa Bio, Shiga, Japan) and 100 mg of lysing enzymes from *Trichoderma harzianum* and incubated on a tube rotator at 28°C for 3 h. After centrifugation (900 × *g*, 10 min, 4°C), the obtained precipitates containing protoplasts were resuspended in 3 mL of a breaking buffer (250 mM sucrose, 20 mM Tris-HCl [pH 7.4], 1 mM EDTA, and 1% protease inhibitor cocktail), and homogenized manually on ice. This suspension was separated to supernatant (containing mitochondria) and pellet (containing nuclei/unbroken cells) fractions by centrifugation (900 × *g*, 10 min, 4°C).

The pellet was incubated with 500 μL of TNE buffer (1% NP-40, 20 mM Tris-HCl [pH 7.4], 1 mM EDTA, 150 mM NaCl and 1% protease inhibitor cocktail) on a test tube rotator for 1 h at 4°C and centrifuged (15,000 × *g*, 10 min, 4°C) to obtain the supernatant, which was used as the nuclear/unbroken cell fraction. Mitochondria-containing supernatant was centrifuged (10,000 × *g*, 10 min, 4°C) to precipitate mitochondria. The resulting supernatant was used as the cytosolic protein fraction. The mitochondria-containing pellet was treated with 100 μL of TNE buffer for 1 h at 4°C. After centrifugation (15,000 × *g*, 10 min, 4°C), the obtained supernatant was used as the mitochondrial protein fraction. The protein concentration was determined with the BCA Protein Assay Reagent (Thermo Fisher Scientific, Waltham, USA), with bovine serum albumin being used as a standard.

### Western blotting

Proteins were separated on 12% polyacrylamide gel and transblotted onto a PVDF membrane. The membrane was blocked with 5% skim milk in TBS-T (50 mM Tris-HCl [pH 7.6], 150 mM NaCl, and 0.05% Tween-20) for 1 h at RT. After washing with TBS-T, the membrane was incubated with TBS-T containing mouse monoclonal anti-cytochrome c antibody (ab110325; Abcam, Cambridge, MA) at a dilution of 1:2000 for 2 h at RT. After washing with TBS-T, the membrane was incubated with TBS-T containing secondary peroxidase-conjugated goat polyclonal anti-mouse IgG (H+L) antibody (Thermo Fisher Scientific) at a dilution of 1:2000 for 1 h at RT. After washing with TBS-T, the membrane was treated with ECL Prime Western Blotting Detection Reagent (GE Healthcare), according to the manufacturer’s protocol. Chemiluminescent signal bands were visualized with an ImageQuant LAS 4000 (GE Healthcare).

### Preparation of proteins from *S*. *cerevisiae*

One milliliter of an overnight-preculture broth of *S*. *cerevisiae* strain INVSc1 (Invitrogen, Waltham, USA) was added to 1 L of YPD medium (1% yeast extract, 2% polypeptone, and 2% D-glucose) and grown to a final optical density at 600 nm (OD_600_) of 1.0 at 26.5°C. Cells were harvested by centrifugation (900 × *g*, 10 min) and resuspended in 50 mL of Tris-SO_4_ buffer (100 mM Tris-SO_4_ [pH 9.4] and 10 mM DTT), followed by incubation for 30 min at 30°C. After centrifugation (900 × *g*, 10 min), the obtained cells were washed with sorbitol buffer (20 mM potassium phosphate [pH 7.4] and 1.2 M sorbitol) and incubated with 50 mL of sorbitol buffer containing 50 mg of zymolyase-20T for 1 h at 30°C to obtain spheroplasts. Spheroplasts were harvested by centrifugation (900 × *g*, 10 min, 4°C), washed once with sorbitol buffer, resuspended in 15 mL of a breaking buffer (20 mM Tris-HCl [pH 7.4], 0.6 M sorbitol, and 1% protease inhibitor cocktail), and homogenized manually on ice. Fractionations were performed by the same procedure as described for *F*. *graminearum*.

### Affinity purification with precocene II-immobilized beads

Affinity purification was carried out according to the manufacturer’s instructions (FG Beads) with slight modifications. Briefly, precocene II-immobilized beads (0.2 mg) were washed three times with a bead-binding buffer (100 mM KCl, 20 mM HEPES-NaOH [pH 7.9], 1 mM MgCl_2_, 0.2 mM CaCl_2_, 0.2 mM EDTA, 10% v/v glycerol, and 0.1% NP-40). Beads and 500 μg of protein extract from each fraction described above were incubated in 300 μL of bead-binding buffer at 4°C for 4 h. Beads were collected magnetically and washed three times with 200 μL of the bead-binding buffer. Bound protein was eluted with 200 μL of bead-binding buffer containing 5 mM precocene II. In competitive inhibition experiments, each protein extract was incubated with 300 μL of bead-binding buffer containing 5 mM precocene II at 4°C for 1 h before incubation with beads.

Each protein solution, after washing three times and eluting with precocene II, was mixed with 600 μL of water, 800 μL of methanol, and 200 μL of chloroform. After centrifugation of the mixture (15,000 × *g*, 2 min), the upper layer was removed, and 800 μL of methanol were added to the remaining lower layer. After vortexing and centrifugation (15,000 × *g*, 2 min), the supernatant was discarded, and the residual pellet was dried. The residue was dissolved in 40 μL of a SDS sample buffer (2% SDS, 5% sucrose, 100 mM DTT, 62.5 mM Tris-HCl [pH 7.4], and 0.002% bromophenol blue) and heated at 100°C for 5 min. The protein sample was subjected to SDS-PAGE on 10% polyacrylamide gels. Proteins were stained with the SilverQuest Silver Staining Kit (Invitrogen).

### In-gel digestion and protein identification by LC/MS/MS

The protein band on SDS-PAGE described above was excised from the gel, destained according to the protocol for the SilverQuest Silver Staining Kit, dehydrated with acetonitrile, and dried. The residue was treated with 50 μL of a reducing solution (10 mM DTT and 100 mM NH_4_HCO_3_) for 1 h at 56°C and 50 μL of an alkylating solution (55 mM iodoacetamide and 100 mM NH_4_HCO_3_) for 45 min at RT in the dark. The residue was further treated successively with 100 mM NH_4_HCO_3_ (100 μL) for 10 min, acetonitrile (100 μL) for 15 min, 100 mM NH_4_HCO_3_ (100 μL) for 10 min, and acetonitrile (100 μL) for 15 min and dried. Proteins in the gel were digested overnight at 37°C with 10 μL of 10 μg/mL Trypsin Gold (Promega, Fitchburg, USA) in 50 mM NH_4_HCO_3_.

The liquid portion of the reaction mixture was transferred to a new tube. The remaining gel residue was extracted three times with 20 μL of a water solution containing 50% acetonitrile and 5% formic acid. The latter extracted solution was pooled with the former liquid portion and dried. Residual pellet was dissolved in 20 μL of a water solution containing 2% acetonitrile and 0.1% formic acid, and was vortexed for 5 min. After centrifugation (15,000 × *g*, 5 min), the supernatant was transferred into an analytical vial, and LC/MS/MS analysis was performed in an Orbitrap Elite system (Themo Fisher Scientific).

Proteome Discoverer 1.4 (Thermo Fisher Scientific) was used for protein identification. *F*. *graminearum* PH-1 (FG3) protein data from the Fusarium Comparative Sequencing Project of the Broad Institute of Harvard and MIT (http://www.broadinstitute.org/) and protein translations of open-reading frame (ORF) data from The Saccharomyces Genome Database (SGD, http://www.yeastgenome.org) were used as protein databases. The protein score, sequence of matched peptides, and percentage of sequence coverage for each identified protein are listed in S1 Table, S2 Table, S3 Table.

### In-vitro binding assay using recombinant VDAC

Recombinant *F*. *graminearum* VDAC containing His_6_ at the N-terminus was prepared according to the method of Bay et al. [38], with some modifications. First-strand cDNA was prepared by the procedure described in the **RT-qPCR analysis** section. The following primers were used for cloning: *VDAC*-F (5’-GGGCCATGGG ATCTGTCCCC GCCTTCTC-3’) and *VDAC*-R (5’-GGGGTCGACT TAACCCTCGA AGGTGAAGC-3’), containing the *Nco*I and *Sal*I sites, respectively. PCR was performed with cDNA as a template. PCR products were cloned into pGEM-T (Easy) Vectors (Promega). After digestion with *Nco*I and *Sal*I, the insert was ligated into the *Nco*I/*Sal*I site of the pPRO EX HTc expression plasmid (Invitrogen).

*Escherichia coli* Rosetta-gami 2 (DE3) cells (TaKaRa Bio) were transformed with the pPRO-based expression plasmid. Transformed cells were cultured overnight in LB medium containing ampicillin (50 μg/mL) at 37°C. Twenty milliliters of culture were inoculated into LB medium (1 L) containing ampicillin (50 μg/mL) and grown to a final OD_600_ of 0.4 at 37°C. IPTG (1 mM) was added to the culture and incubated for an additional 3 h at 37°C. Bacterial cells were harvested by centrifugation, resuspended in 50 mL of PBS (10 mM potassium phosphate [pH 7.4] and 150 mM NaCl) and disrupted by sonication. After centrifugation (2,500 × *g*, 15 min, 4°C), residual pellets containing the rHis-VDAC protein were washed twice with PBS containing 1% Triton X-100 and solubilized in 3 mL of a denaturation buffer (8 M urea, 20 mM HEPES-KOH [pH 7.6], 500 mM NaCl and 0.1% NP-40) containing 10 mM imidazole. The rHis-VDAC protein was purified with a Ni Sepharose 6 Fast Flow affinity resin column (GE Healthcare, Little Chalfont, UK), according to the manufacturer’s protocol. Then, rHis-VDAC was eluted with denaturation buffer containing 500 mM imidazole. After dialysis against the denaturation buffer, rHis-VDAC was precipitated with cold acetone. Obtained precipitates were dried and resolubilized in 1 mL of a SDS/DDM solution (3.5 mM SDS and 30 mM dodecyl-β-D-maltoside). The protein concentration was determined with BCA Protein Assay Reagent.

Five micrograms of the rHis-VDAC protein were mixed with 300 μL of the binding buffer described above and incubated with the precocene II-immobilized magnetic beads or epoxy beads as a control. Beads were washed with binding buffer three times. Bound protein was eluted with 50 μL of SDS sample buffer at 100°C for 5 min. The solution containing unbound rHis-VDAC obtained after removing the beads was pooled with the washing solutions and used as the flow-through sample.

After electrophoresis of the flow-through and eluted samples on 10% polyacrylamide gel, proteins on the gel were transblotted onto a PVDF membrane. The subsequent procedure was the same as that described in **Western blotting,** except that mouse monoclonal anti-N-terminus 6 × Histidine antibody (Biodynamics Laboratory Inc., Tokyo, Japan) at a dilution of 1:1000 was used as primary antibody.

### Microscopy

MitoSOX (5 μM, Molecular Probes, Waltham, USA) in Hanks’ Balanced Salt Solution (HBSS, Gibco, Waltham, USA) was prepared according the manufacturer’s instructions and used for detection. DHE (30 mM) dissolved in anhydrous DMSO was diluted 1,000-fold with HBSS and used for detection. *F*. *graminearum* was cultured in SYEP liquid medium (5 mL) in a test tube for 24 h at 26.5°C. Methanolic precocene II (1.5 μL) or aqueous paraquat (5 μL) was added to the culture and incubated for an additional 24 h at 26.5°C. Mycelia were harvested by filtration, washed with water, and incubated with HBSS containing 5 μM mitoSOX or 30 μM DHE for 10 min at 37°C in the dark. Mycelia were washed with HBSS and applied to microscope slides.

To obtain images, a BX53 (Olympus, Tokyo, Japan) fluorescence microscope equipped with DP70 camera (Olympus) was used. The U-FBW fluorescence filter cube (Olympus) was used for fluorescence of both mitoSOX and DHE. The camera exposure time was set at 1 s for both mitoSOX and DHE fluorescence. For rough quantification of mitochondrial superoxide in fungal cells, *F*. *graminearum* was cultured in SYEP liquid medium (5 mL) in a test tube with precocene II (30 μM) for 24–96 h at 26.5°C. After incubation, mycelia were harvested by filtration, washed with water, incubated with HBSS containing 5 μM mitoSOX for 10 min at 37°C in the dark, and incubated with HBSS containing 3 μM Fluorescent Brightener 28 for 2 min at 37°C. Mycelia were washed once with HBSS and applied to microscope slides. The U-FUW fluorescence filter cube was used for fluorescence of Fluorescent Brightener 28, and the camera exposure time was set at 5 ms.

For quantitative analysis, Image J software (US National Institutes of Health, Bethesda, USA) was used. To estimate the mycelial mass in an image, each microscopic fluorescent image of Fluorescent Brightener 28 was split into three color components (blue, red, and green). Because Fluorescent Brightener 28 exhibited blue fluorescence under the U-FUW filter, the blue component was used for further analysis. To obtain a binarized image, a grey-level threshold was applied at “Auto”. The dimension of the black area in the resulting binarized image was analyzed and used as the estimated mycelial mass.

To calculate the amount of superoxide in an image, a microscopic image of mitoSOX fluorescence was subjected to background subtraction (rolling ball radius = 300 px) and split into three color components. In the red component, a grey-level threshold was applied at “20”. The dimension of the black area in the resulting binarized image was analyzed and used as the estimated superoxide area. The relative superoxide level was calculated with the equation: superoxide in an image = estimated superoxide area/estimated mycelial mass × 100. The value of cells cultivated for 24 h without precocene II was used as the control.

### RT-qPCR analysis

*F*. *graminearum* was cultured in SYEP liquid medium (5 mL) in a test tube with or without precocene II (30 μM) for 24–96 h at 26.5°C. After incubation, mycelia were harvested by filtration and lyophilized. Total RNA was extracted and purified with TRIzol reagent (Invitrogen) and the PureLink RNA Mini Kit (Ambion, Waltham, USA), respectively, according to the manufacturer’s protocol. The cDNA was prepared with the ReverTra Ace qPCR RT Master Mix (TOYOBO, Osaka, Japan), according to the protocol. The cDNA derived from 0.05 μg of total RNA was used as a template.

RT-qPCR was carried out by using FastStart Universal SYBR Green Master (Rox) (Roche, Basel, Switzerland) in a final volume of 15 μL for each reaction and an ABI PRISM 7300 thermal cycler (Applied Biosystems, Waltham, USA). Two-step PCR conditions were as follows: after an initial incubation at 95°C for 10 min, 40 cycles of 95°C for 15 s and 60°C for 1 min were performed. The amount of *Tri6* mRNA was normalized to the amount of *GAPDH* mRNA (control gene) in each sample. PCR primers used for each gene were as follows: *Tri6*-F (5’-TGCTCGGCAT GAGTCTAAGC-3’), *Tri6*-R (5’-CACCGATCCC TCGTCAACAC-3’), *GAPDH*-F (5’-TCAAGGGTGT TCTGGCCTAC-3’), and *GAPDH*-R (5’-AGTAACCCCA CTCGTTGTCG-3’).

### Mitochondrial protein carbonyl content

*F*. *graminearum* was cultured in SYEP liquid medium (100 mL) in a 500-mL Erlenmeyer flask with or without precocene II (30 μM) for 36 h at 26.5°C. Mycelia were harvested by filtration, resuspended in 5 mL of 1.5 M aqueous NaCl solution containing 100 mg of Yatalase and 100 mg of lysing enzymes from *Trichoderma harzianum*, and incubated on a tube rotator at 28°C for 3 h. After centrifugation (900 × *g*, 10 min), the precipitates containing protoplasts were resuspended in 3 mL of a breaking buffer (250 mM sucrose, 20 mM HEPES-KOH [pH 7.2], 10 mM DTT, 1 mM EDTA, and 1% protease inhibitor cocktail), and homogenized manually on ice. After centrifugation (900 × *g*, 10 min, 4°C), the supernatant was recovered and further centrifuged (10,000 × *g*, 15 min, 4°C) to obtain mitochondria. The mitochondria-containing pellet was resuspended in 200 μL of RIPA buffer (1% NP-40, 0.1% sodium deoxycholate, 0.1% SDS, 50 mM HEPES-KOH [pH 7.2], 1 mM EDTA, 150 mM NaCl, and 1% protease inhibitor cocktail) and incubated for 10 min at RT. Streptomycin sulfate was added to a final concentration of 1% for precipitation of nucleic acids, and the solution was allowed to stand for 10 min at RT. After centrifugation (10,000 × *g*, 5 min), the supernatant was recovered and the protein concentration was determined. Mitochondrial protein carbonyl content was measured as described by Dalle-Donne et al. [27], with some modifications. Briefly, 200 μg of mitochondrial protein was mixed with 2 M HCl solution containing 10 mM 2.4-dinitrophenylhydrazine (DNPH) to a final concentration of 2 mM and allowed to stand for 1 h at RT, with vortexing every 15 min. Trichloroacetic acid (TCA) was added to the solution at a final concentration of 20%, and the mixture was incubated on ice for 10 min. Protein precipitates were collected by centrifugation, washed once with 20% TCA, washed three times with ethanol/ethyl acetate (1:1), and solubilized in 200 μL of a 2 M HCl solution containing 6 M guanidine hydrochloride. Absorbance of the solution at 366 nm was measured, and carbonyl contents were calculated by using a molar absorption coefficient of 22,000 M^-1^ cm^-1^. Protein concentration of the solution was determined with BCA Protein Assay Reagent, and the amount of incorporated DNPH per mg of protein was calculated.

### Statistical analysis

Data are presented as the mean + standard deviation (SD). Differences between more than three groups were assessed by one-way ANOVA followed by the Dunnett test. Differences between two means were determined with the Welch *t*-test. Values of *P* < 0.05 were considered to be significant.

## Supporting Information

S1 Fig Detection of cytochrome c. Protoplasts of *F*. *graminearum* were fractionated to nuclear/unbroken cell, mitochondrial, and cytosolic fractions. Cytochrome c was detected by immunoblotting. Protein extract (10 μg) was separated in each lane.
(TIF)

S1 Scheme Preparation of 6-*O*-demethylprecocene II and precocene II-immobilized beads. (A) Preparation of 6-*O*-demetylprecocene II. (B) Preparation of precocene II-immobilized beads.
(TIF)

S1 Table Proposed proteins as precocene II-binding protein from peptide sequences determined by LC/MS/MS from mitochondrial fraction of *F*. *graminearum*. (XLSX)

S2 Table Proposed proteins as precocene II-binding protein from peptide sequences determined by LC/MS/MS from nuclear/unbroken cell fraction of *F*. *graminearum*. (XLSX)

S3 Table Proposed proteins as precocene II-binding protein from peptide sequences determined by LC/MS/MS from yeast mitochondrial fraction. (XLSX)
