# Supplementary figures and images for: Precocene II, a Trichothecene Production Inhibitor, Binds to Voltage-Dependent Anion Channel and Increases the Superoxide Level in Mitochondria of Fusarium graminearum
Source: PLoS One. 2015 Aug 6;10(8):e0135031. doi: 10.1371/journal.pone.0135031 (PMC4527739; doi:10.1371/journal.pone.0135031)

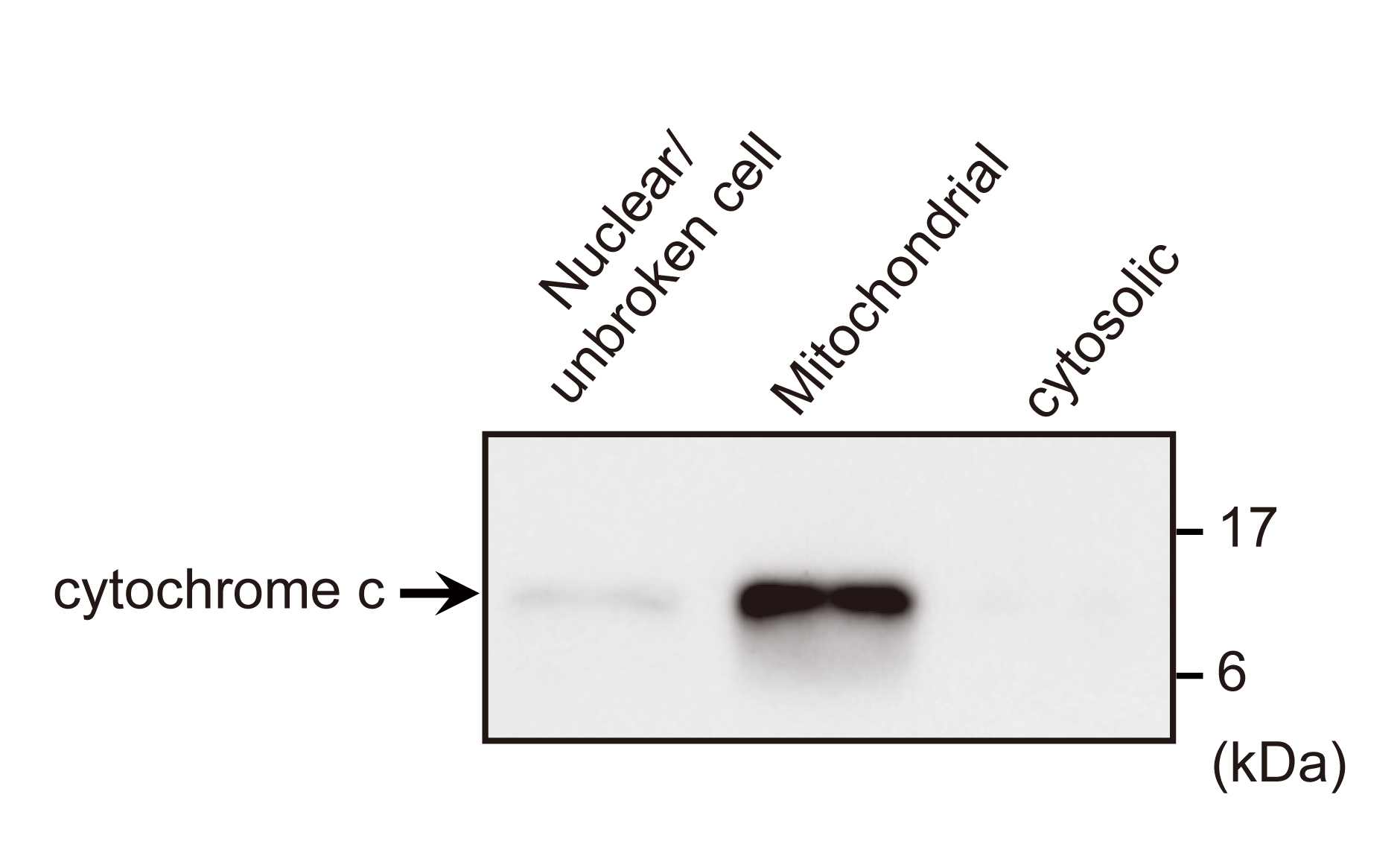

Supplement: S1 Fig — Protoplasts of F. graminearum were fractionated to nuclear/unbroken cell, mitochondrial, and cytosolic fractions. Cytochrome c was detected by immunoblotting. Protein extract (10 μg) was separated in each lane. (TIF) [file pone.0135031.s001.tif]
